# Accelerating the Switchgrass (*Panicum virgatum* L.) Breeding Cycle Using Genomic Selection Approaches

**DOI:** 10.1371/journal.pone.0112227

**Published:** 2014-11-12

**Authors:** Alexander E. Lipka, Fei Lu, Jerome H. Cherney, Edward S. Buckler, Michael D. Casler, Denise E. Costich

**Affiliations:** 1 Institute for Genomic Diversity, Cornell University, Ithaca, New York, United States of America; 2 Department of Crop and Soil Sciences, Cornell University, Ithaca, New York, United States of America; 3 Agricultural Research Service, United States Department of Agriculture, Ithaca, New York, United States of America; 4 Department of Plant Breeding and Genetics, Cornell University, Ithaca, New York, United States of America; 5 Agricultural Research Service, United States Department of Agriculture, Madison, Wisconsin, United States of America; 6 Department of Agronomy, University of Wisconsin–Madison, Madison, Wisconsin, United States of America; USDA-ARS-SRRC, United States of America

## Abstract

Switchgrass (*Panicum virgatum* L.) is a perennial grass undergoing development as a biofuel feedstock. One of the most important factors hindering breeding efforts in this species is the need for accurate measurement of biomass yield on a per-hectare basis. Genomic selection on simple-to-measure traits that approximate biomass yield has the potential to significantly speed up the breeding cycle. Recent advances in switchgrass genomic and phenotypic resources are now making it possible to evaluate the potential of genomic selection of such traits. We leveraged these resources to study the ability of three widely-used genomic selection models to predict phenotypic values of morphological and biomass quality traits in an association panel consisting of predominantly northern adapted upland germplasm. High prediction accuracies were obtained for most of the traits, with standability having the highest ten-fold cross validation prediction accuracy (0.52). Moreover, the morphological traits generally had higher prediction accuracies than the biomass quality traits. Nevertheless, our results suggest that the quality of current genomic and phenotypic resources available for switchgrass is sufficiently high for genomic selection to significantly impact breeding efforts for biomass yield.

## Introduction

Switchgrass (*Panicum virgatum* L.) is undergoing development as a biofuel feedstock due to its high biomass yield, broad adaptation, perennial growth habit, and long-standing presence in the seed industry [1]. Once inhabiting prairie and savanna ecosystems from Canada to Mexico and east of the Rocky Mountains, native switchgrass is now confined to thousands of prairie and savanna remnants that range in size from a few plants to a few hundred hectares [2]. Driven largely by photoperiod and temperature, latitude is the principal source of adaptive phenotypic variability across a broad landscape [3], [4].

Switchgrass contains three principal taxa: a tetraploid (2n = 4x = 28) lowland ecotype, a tetraploid upland ecotype, and an octoploid (2n = 8x = 56) upland ecotype [5]. Upland ecotypes originated from upland prairie and savanna habitats that were frequently exposed to drought, especially toward the western portion of the range [6]. Lowland ecotypes originated in low-lying riverine or lacustrine habitats that were exposed to seasonal wet periods [6]. Upland ecotypes tend to be more northern adapted, while lowland ecotypes tend to be more southern adapted, with a transition zone where both can be found, sometimes within a single prairie or savanna remnant [5]. Upland and lowland ecotypes are highly cross-fertile and significant gene flow has occurred between the ecotypes during glacial maxima of the past million years [7]. Ploidy is the secondary taxonomic division within the species, primarily within the upland ecotype; lowland plants at the octoploid level are rare [8]. Gene flow has occurred between tetraploid and octoploid levels, largely by 2n gametes (4x to 8x) or haploidy (8x to 4x), but at relatively low frequencies due to the role of ploidy as a hybridization barrier [9].

Since the establishment of switchgrass as the herbaceous model species for cellulosic biofuel feedstock development in 1992 [1], a total of 12 breeding programs have been developed in North America [5]. Due to phenotypic differences among the three principal taxa and to the magnitude of adaptive phenotypic variation for flowering time and temperature (cold and heat) tolerance, there is very little overlap or duplication among these breeding programs. Collectively, their target population of environments covers eastern North America, but their individual target regions are realistically broken down into a minimum of eight regional gene pools or cultivar deployment zones [5]. Because adaptive phenotypic variation is a strong driver of both adaptation and production traits, genotype × environment interactions are a dominant force and individual cultivars are rarely adapted to more than three hardiness zones, as defined by [10].

Due to the length of the breeding cycle and the need for frequent (perhaps constant) phenotypic assessment of adaptive traits, few cultivars have been developed with documented improvement in biomass production traits. ‘Liberty’ is the most notable example, demonstrating both an increase in biomass yield and broader adaptation into USDA hardiness zone 3 [11]. Recent advances in the development of genomic tools for measuring and quantifying DNA marker diversity and sophisticated statistical tools to associate marker variation with phenotypic variation have the potential to revolutionize switchgrass breeding methodology [12]. Switchgrass breeding is complicated by the perennial nature of the species and the need for accurate measurement of biomass yield on a per-hectare basis, the single trait that is most limiting for sustainable and economically viable biomass production [13]. Simple-to-measure surrogate traits are needed to speed up the breeding cycle. Genomic selection [14], [15] offers such an opportunity by developing predictive equations that allow breeders to measure DNA markers on seedlings and to predict which seedlings will have the highest biomass yield potential as adult plants [12].

The potential of genomic selection for improving the effectiveness of breeding programs has been successfully demonstrated in livestock [16]–[18], annual crops [19]–[23], and forest trees [24]–[26]. In these species, genomic selection has been shown to increase selection accuracy, reduce evaluation cost per genotype, and reduce breeding cycle time compared to phenotypic selection. More specifically, a recent evaluation of genomic selection methods concluded that genomic selection for perennial biofuel crops, such as switchgrass, is most advantageous when biomass yield on a per-hectare basis is difficult or expensive to measure, when it is difficult or impossible to apply meaningful selection pressure on plants within families, and when cycle times are >5 years, which is typically the case with switchgrass [12].

The purpose of this study was to explore the potential for genomic selection to increase the breeding cycle in switchgrass, particularly for seven morphological traits and 13 biomass quality traits. For most of these traits, reasonably high prediction accuracies were obtained. Our analysis was conducted within an association panel of 515 genotypes defined as a random sample of switchgrass from the northern USA gene pools. The population was evaluated using a set of 16,669 single nucleotide polymorphisms (SNPs) obtained using genotyping by sequencing (GBS) techniques [27], [28] that were subsequently mapped to the recently available *Panicum virgatum* genome sequence v1.1 reference genome [29].

## Materials and Methods

### Germplasm

We analyzed the switchgrass association panel described in [27]. Briefly, this panel included 66 diverse switchgrass populations derived from predominantly northern adapted upland germplasm. Both tetraploid and octoploid germplasm were included. This panel was grown from seed planted at the greenhouse in the USDA-ARS Dairy Forage Research Center in Madison, Wisconsin in 2007. Ten clones or genotypes from each population were vegetatively propagated, then planted in Ithaca, New York in 2008 in a randomized complete block design with two replicates. Subsequently, a total of 540 plants from the Ithaca location were used for genotypic and phenotypic evaluation.

### Morphological traits

The association panel was evaluated for seven morphological traits in 482 of the plants grown in Ithaca, NY during the 2009, 2010, and 2011 field seasons. These traits included anthesis date, heading date, standability, leaf length, leaf width, plant height, and total plant height. Descriptions of how each of these traits was measured are presented in Table 1, and the tools used to obtain the measurements are described at http://www.maizegenetics.net/phenotyping-tools [30]. Prior to subsequent analysis, the heading and anthesis dates were converted to growing degree days (GDD) as follows:

**Table 1:** Phenotyping protocol for seven morphology traits measured in three summer environments, in Ithaca, NY across three years.

Trait Name (units)	Trait Description	Measured in Following Years
Anthesis Date	50% of panicles have 50% open florets	2009–2011
Heading Date	at least 50% of stems are 50% emerged (panicle branches still upright, just starting to spread)	2009–2011
Standability	0 = prostrate	2010–2011
	10 = upright	
Leaf length (mm)	Leaf below flag; base to tip	2009–2011
Leaf width (mm)	Leaf below flag; widest part	2009–2011
Plant Height (cm)	Base of longest flowering stem to the node at the base of the panicle	2009–2011
Total Plant Height (cm)	Base of the longest flowering stem to the tip of the panicle	2009–2011

-

1. The first day in which GDD was recorded occurs the day after the first five consecutive days where the average temperature is >32° F.
2. After this day, GDD for a single day is calculated as: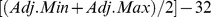 where *Adj. Min* is the maximum of the minimum daily temperature and 32°F, and *Adj.Max* is the minimum of the of the maximum daily temperature and 86°F. Intuitively, *Adj. Min* and *Adj.Max* limits the recorded minimum and maximum daily temperatures to 32°F and 86°F, respectively.
3. For each day after the first day in which GDD is recorded, the cumulative GDD is also recorded. The cumulative GDD is used to record heading date and anthesis date.

### Biomass Quality traits

Near-infrared reflectance spectroscopy (NIRS, described in [31]) was used to estimate 42 biomass quality traits for a total 515 genotypes grown during two field seasons at the Ithaca, NY location. Samples were ground in Ithaca, NY, shipped to Madison, WI, and scanned on an NIRS unit at the U.S. Dairy Forage Research Center, as described in [31]. A total of 42 biomass quality traits were predicted using equations developed by [31], but only 13 of those traits were analyzed in this study due to their direct relevance and practical value in a breeding program focused on improving conversion efficiency, and to minimize redundancy from collinear traits. Specifically, these traits include acid detergent lignin, minerals (total ash), carbon, high heating value, cell wall concentration, ethanol/g dry forage, etherified ferulates, in vitro dry matter digestibility, pentose sugars release/g dry forage, total soluble carbohydrates, starch, sucrose, and total sugar. No sample had an H-statistic >3.0, indicating that none of the samples could be classified as outliers.

### Description of SNPs

The Universal Network-Enabled Analysis Kit (UNEAK) discovery pipeline [27] was used to generate 29,221 SNPs with a minimum call rate of 0.5 and minimum minor allele frequency (MAF) of 0.05 among the 540 plants grown at the Ithaca location. These SNPs were then aligned to the *Panicum virgatum* genome sequence v1.1 [29]. The resulting 16,669 uniquely aligned SNPs were used for subsequent analysis.

### Phenotypic evaluation

A subset of the 540 plants that yielded sufficient biomass for at least one field season was evaluated for morphological and quality traits. Specifically, 482 plants were evaluated for seven morphological traits and 515 plants were evaluated for 13 quality traits. All 20 traits were examined for outliers using Studentized deleted residuals [32] from a mixed linear model including year, field, block, and population as random effects in SAS version 9.3 [33]. For each trait, best linear unbiased predictors (BLUPs) were obtained for each line across years and replicates, using a mixed linear model fitted in ASReml version 3.0 [34]. Details of the model fitting procedure have been described in [35]. The relationship between each of these BLUPs was then evaluated using the Pearson correlation coefficient (*r*). Variance component estimates from the model used to obtain BLUPs were also used to estimate repeatability on a clone mean basis (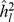) [36], [37]. These repeatability estimates are upper bounds of the heritabilities for each trait. The delta method was used to approximate the standard error of the repeatability estimates [36]. Finally, the Box-Cox procedure [38] was implemented to find the optimal transformation of the BLUPs, as described in [39].

### Genomic Selection

Prior to evaluating the genomic selection models, missing allelic values among the 16,669 SNPs anchored to the *Panicum virgatum* genome sequence v1.1 reference genome were imputed using fastPhase version 1.4.0 [40]. The allele frequencies of these SNPs were calculated among the 482 plants evaluated for the morphology traits and again among the 515 plants evaluated for the quality traits. Within each of these two subsets, SNPs with MAF <0.05 were removed. Consequently, 11,857 SNPs were used in the genomic selection models for the morphology traits, and 12,180 SNPs were used in the models for the quality traits.

To assess the capability of our imputed markers to predict morphological and quality trait values, three genomic selection approaches were tested, namely ridge regression-best linear unbiased prediction (RR-BLUP) [14], least absolute shrinkage and selection operator (LASSO) [41], and elastic net [42]. Although these three approaches have been shown to produce similar results in practice (e.g., [21]), the performance of each approach could depend on the genetic architecture of the evaluated traits. Specifically, RR-BLUP should theoretically outperform LASSO for complex traits, while LASSO should be superior for simpler traits. The elastic net, whose penalty is a weighted average of the penalties of RR-BLUP and LASSO, is considered to be a compromise between the two approaches. In this study, the mixing parameter for the elastic net was set to α = 0.5, meaning that the RR-BLUP and LASSO penalties were given equal weights. The RR-BLUP approach was conducted using the rrBLUP package [43] in the R programming language [44], while LASSO and elastic net were conducted using the glmnet R package [45].

For any genomic selection model, it is important to ensure that SNP effects arising from overall differences in population structure are factored out [17]. Given the genetic differences attributable to the observed ecotypes and ploidies in our association panel, it is crucial to account for such SNP effects prior to conducting our genomic selection study. Based on the results presented in [27], we hypothesized that the first two principal components (PCs) of a principal component analysis (PCA) of the 16,669 SNPs imputed with fastPhase would sufficiently account for these genetic differences. Accordingly, we fitted a model to each trait where the trait was the response variable and the first two PCs from the PCA of these SNPs were the explanatory variables. The residuals from each model were used for genomic selection.

The performance of each model was assessed through ten-fold cross validation, as described in [46]. Briefly, the association panel was partitioned into ten equally-sized subgroups. Nine of the ten subgroups (i.e., the training set) were used to fit each prediction model while the remaining subgroup (the prediction set) was used to assess the correlation between the observed and predicted trait values. This process was repeated ten times, with each subgroup being the prediction set exactly once. For each trait, prediction accuracies were calculated by dividing the average Pearson's correlation coefficient across the ten folds by the square root of the repeatability [25]. To prevent inflated prediction accuracies arising from clones nested within populations, the data were partitioned for ten-fold cross validation so that none of the populations were in both the training and prediction sets. All phenotypic and genotypic data used to conduct this analysis are included in File S1.

## Results

### Extensive Phenotypic Variability among Clones

Substantial variation was observed for each of the seven morphological traits, with differences between minimum and maximum values of each trait ranging from 2-fold for anthesis date to 5.84-fold for standability (Table 2). In general, the majority of the morphological traits were highly correlated, with the strongest Pearson's correlation being between heading date and anthesis date (*r* = 0.92; Table S1). High correlations between leaf width, plant height, and total plant height were also observed (Pearson correlations ranging from *r* = 0.53 to *r* = 0.88). The average repeatability among the seven morphological traits was 0.86, with a range from 0.75 for plant height to 0.93 for anthesis date (Table 2). These high repeatabilities suggest that the majority of the phenotypic variation is attributable to genetic effects, and that genomic selection could be a useful breeding approach for morphological traits in switchgrass.

**Table 2:** Means and ranges for best linear unbiased predictors (BLUPs) of seven morphological traits evaluated on a switchgrass association panel, and estimated repeatability on a clone-mean basis in three summer environments, in Ithaca, NY across three years.

Trait	No. Lines	BLUP Mean	BLUP SD^b^	BLUP Range	Repeatability	Repeatability SE^c^
Anthesis Date (GDD^a^)	481	3840.53	450.21	2630.25–5272.48	0.93	0.01
Heading Date (GDD)	482	2870.47	343.81	2111.75–4547.04	0.91	0.01
Standability (0–10 scale)	481	5.36	1.60	1.47–8.59	0.88	0.01
Leaf Length (mm)	482	528.88	73.30	294.62–708.48	0.85	0.02
Leaf Width (mm)	482	13.32	1.91	6.56–20.75	0.82	0.02
Plant Height (cm)	482	88.78	16.45	44.75–146.16	0.75	0.03
Total Plant Height (cm)	482	162.22	20.24	105.43–222.81	0.85	0.02

a GDD, Growing degree dates

b SD, Standard deviation

c SE, Standard error

In comparison to the morphological traits, a greater range of fold differences between the minimum and maximum values of each trait was observed for the quality traits (Table 3). Although many of the correlations between the quality traits were generally lower than those between the morphology traits, some individual quality traits were strongly correlated. For example, a Pearson correlation coefficient of *r* = 0.95 was observed between sucrose and total soluble carbohydrates (Table S2). Although lower than observed among the morphology traits, the estimated repeatabilities of the quality traits were sufficiently high enough to merit investigation into the utility of genomic selection.

**Table 3:** Means and ranges for best linear unbiased predictors (BLUPs) of 13 quality traits evaluated on a switchgrass association panel, and estimated repeatability on a clone-mean basis in two summer environments, in Ithaca, NY, across two years.

Trait (µg/g)	No. Lines	BLUP Mean	BLUP SD^a^	BLUP Range	Repeatability	Repeatability SE^b^
Acid detergent lignin	514	75.62	5.45	61.47–90.02	0.81	0.02
Minerals (total ash)	514	69.20	4.60	54.47–83.62	0.67	0.03
Carbon	514	443.86	2.19	438.78–452.85	0.67	0.03
High Heating Value	514	4182.59	17.6	4136.14–4237.54	0.76	0.02
Cell wall concentration	514	673.11	47.31	564.12–832.92	0.87	0.01
Ethanol/g dry forage	514	82.73	7.46	60.83–106.78	0.78	0.02
Etherified ferulates	514	0.88	0.10	0.64–1.28	0.83	0.48
In vitro dry matter digestibility	514	410.54	35.16	311.86–494	0.82	0.01
Pentose sugars release/g dry forage	515	191.29	8.30	167.13–218.05	0.77	0.02
Total soluble carbohydrates	514	51.27	8.58	29.22–74.16	0.71	0.03
Starch	514	6.35	2.72	0.67–17.27	0.59	0.04
Sucrose	514	28.29	5.71	13.71–45.13	0.72	0.02
Total sugar	514	625.44	19.01	572.91–691.56	0.79	0.02

a SD, Standard deviation

b SE, Standard error

### First Two Principal Components of SNPs Sufficiently Account for Ploidy and Ecotype Differences

**Figure 1:**
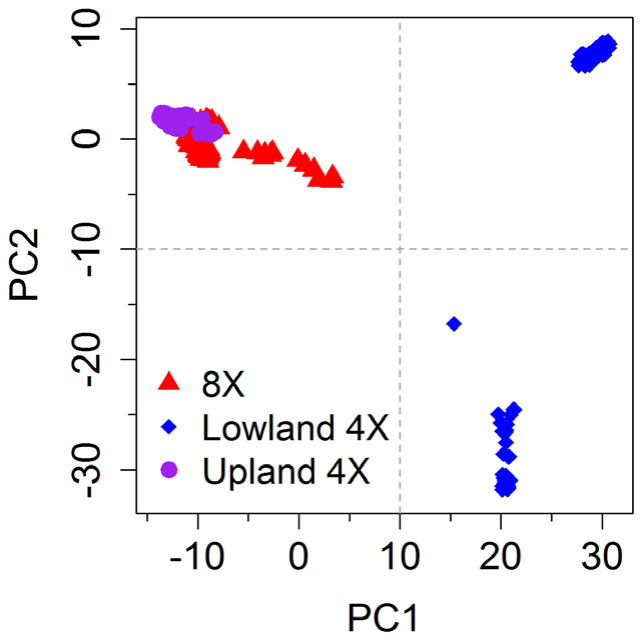
First two principal components of 16,669 single nucleotide polymorphisms separate plants by ploidy and ecotype. The first and second principal components (x- and y-axis, respectively) from a principal component analysis of 540 switchgrass closes separate octoploid (8X) and upland tetraploid (4X) accessions from lowland 4X accessions. The lowland accessions are also separated into two distinct clusters.

The first two PCs of the imputed GBS markers subdivided the plants used in this study into three genetically distinct subgroups (Figure 1). Specifically, the octoploid and upland tetraploid plants were clustered into one group, while the lowland tetraploid plants were subdivided into two distinct clusters. Collectively, these results suggest that the first two PCs of the SNPs capture a substantial amount of the major genetic differences between the ploidies and ecotypes of the plants included in our association panel. Moreover, these results justify our use of the first two PCs to factor out the SNP effects arising from overall population structure differences prior to conducting our genomic selection study.

### Genomic Selection has Considerable Potential in Switchgrass

As expected, the observed prediction accuracies were similar across the three GS approaches (Tables 4–5). The predictive ability of the morphological traits were generally higher than those of the quality traits, with the highest prediction accuracies (averaged across all three tested GS models) obtained for standability (0.52), anthesis date (0.44), ethanol/g dry forage (0.43), leaf length (0.40), and heading date (0.36). We also obtained relatively strong positive Spearman's rank correlation coefficients between repeatabilities and unstandardized prediction accuracies for both the morphology (*r_SP_* = 0.61) and the quality (*r_SP_* = 0.44) traits. Consistent with the findings of previous studies (e.g., [25]), this result suggests that all three GS approaches successfully use the larger genetic contribution to phenotypic variability of the more heritable traits to obtain higher prediction accuracies.

**Table 4:** Prediction accuracies of seven morphological traits in a switchgrass association panel.

Trait	Mean prediction accuracy	Prediction accuracy: RR-BLUP^a^	Prediction accuracy: Elastic net	Prediction accuracy: LASSO^b^
Anthesis Date	0.44	0.55 (0.21)	0.38 (0.23)	0.38 (0.23)
Heading Date	0.36	0.39 (0.14)	0.34 (0.20)	0.34 (0.19)
Standability	0.52	0.51 (0.27)	0.53 (0.19)	0.52 (0.19)
Leaf Length	0.40	0.55 (0.21)	0.34 (0.30)	0.32 (0.29)
Leaf Width	0.19	0.32 (0.29)	0.13 (0.24)	0.13 (0.24)
Plant Height	0.25	0.34 (0.18)	0.21 (0.26)	0.20 (0.26)
Total Plant Height	0.15	0.30 (0.28)	0.09 (0.19)	0.06 (0.19)

Standard errors of prediction accuracies are provided in parentheses.

Mean prediction accuracies were obtained by averaging results across ridge regression best linear unbiased prediction (RR-BLUP), least absolute shrinkage and selection operator (LASSO), and elastic net analysis.

a RR-BLUP, Ridge regression-best linear unbiased prediction

b LASSO, Least absolute shrinkage and selection operator

**Table 5:** Prediction accuracies of 13 quality traits in a switchgrass association panel.

Trait	Mean prediction accuracy	Prediction accuracy: RR-BLUP^a^	Prediction accuracy: Elastic net	Prediction accuracy: LASSO^b^
Acid detergent lignin	0.34	0.41 (0.25)	0.31 (0.21)	0.30 (0.21)
Minerals (total ash)	−0.08	−0.09 (0.18)	−0.06 (0.13)	−0.10 (0.15)
Carbon	0.12	0.21 (0.25)	0.09 (0.27)	0.07 (0.27)
High Heating Value	0.22	0.26 (0.14)	0.21 (0.16)	0.20 (0.17)
Cell wall concentration	0.23	0.30 (0.23)	0.21 (0.19)	0.19 (0.18)
Ethanol/g dry forage	0.43	0.46 (0.20)	0.42 (0.20)	0.41 (0.21)
Etherified ferulates	0.22	0.27 (0.23)	0.20 (0.16)	0.19 (0.15)
In vitro dry matter digestibility	0.35	0.43 (0.27)	0.32 (0.25)	0.30 (0.25)
Pentose sugars release/g dry forage	0.06	0.15 (0.20)	0.03 (0.26)	0.01 (0.25)
Total soluable carbohydrates	0.30	0.39 (0.21)	0.26 (0.23)	0.25 (0.23)
Starch	0.08	0.19 (0.26)	0.03 (0.16)	0.03 (0.15)
Sucrose	0.32	0.44 (0.20)	0.26 (0.24)	0.25 (0.24)
Total sugar	0.04	0.16 (0.17)	0.00 (0.14)	−0.03 (0.17)

Standard errors of prediction accuracies are provided in parentheses.

Mean prediction accuracies were obtained by averaging results across ridge regression best linear unbiased prediction (RR-BLUP), least absolute shrinkage and selection operator (LASSO), and elastic net analysis.

a RR-BLUP, Ridge regression-best linear unbiased prediction

b LASSO, Least absolute shrinkage and selection operator

## Discussion

We evaluated the ability of three popular genomic selection approaches to predict the phenotypic values of seven morphological traits and 13 quality traits in a switchgrass association panel. Such a study is important because the successful application of genomic selection to switchgrass could significantly reduce the breeding cycle of this important biofuel feedstock. In general, our prediction accuracies are comparable to those reported in previous studies (e.g., [17] and [23]) that identified quantifiable advantages of genomic selection compared to traditional breeding programs. For perennial grasses such as switchgrass, one important quantity to consider is the expected genetic gain per unit of time. Because genomic selection does not require on-site phenotyping to identify accessions with superior trait values, multiple cycles of breeding could be completed with a genomic selection breeding program during the same amount of time required to achieve one cycle of breeding using traditional breeding programs [17], [23]. For instance, it is demonstrated in [23] that it is possible for genomic selection breeding programs in maize and winter wheat to respectively achieve three cycles and two cycles of breeding during the same amount of time to complete one cycle of marker-assisted selection breeding. Moreover, the same study concluded that the expected genetic gain per year from a genomic selection breeding program will exceed that of a marker-assisted selection breeding program for traits with prediction accuracies as low as 0.20 in maize and 0.30 in winter wheat. Because many of our tested traits had prediction accuracies that exceed these thresholds, we believe that it is possible for similar advantages in expected genetic gain per unit of time to be achieved in switchgrass genomic selection breeding programs.

To our knowledge, the genetic architectures of the traits we evaluated are unknown in switchgrass. In particular, little is known about the number of genes underlying each trait. Therefore, we used three genomic selection models that have been hypothesized to perform differently under various genetic architectures. In general, we obtained similar prediction accuracies for all three genomic selection models. This result is especially apparent if we consider the standard errors of the prediction accuracies. Suppose we use the prediction accuracies and their standard errors from each genomic selection model (presented in Tables 4 and 5) to construct 95% confidence intervals. For each trait, the confidence intervals from the three genomic selection approaches overlap. This suggests that there are no discernible differences in prediction accuracies among the three genomic selection models. Indeed, this finding has been reported in other studies (e.g. [46]) and is theoretically justified in [47]. Nevertheless, we recommend repeating our study because we anticipate that the sampling, genotyping, and phenotypic resources available to the switchgrass community will continue to expand and improve, and it is imperative to confirm that these conclusions still hold given the new information we expect to obtain from these resources.

We observed a wide range of prediction accuracies across the traits. We suspect that this result was obtained because our markers provided incomplete coverage of the switchgrass genome, and it is likely that they tagged only a subset of the loci underlying the genetic sources of variation for each trait. It is therefore plausible that traits with higher prediction accuracies have causal loci that were in higher linkage disequilibrium with our markers compared to traits with lower prediction accuracies. Nonetheless, the prediction accuracies for many of the studied traits were suitably high enough to justify further investigation into the application of genomic selection to switchgrass breeding programs. Indeed one major factor contributing to our observed prediction accuracies was the availability of the *Panicum virgatum* genome sequence v1.1 reference genome. Because of this reference genome, we were able to use genotypic information from neighboring markers to impute missing genotypic data, and ultimately obtain substantial increases in the predictive abilities of our genomic selection models. Thus, we strongly recommend that switchgrass genomic selection breeding programs only use markers that are anchored to a reference genome. This will enable accurate imputation of missing data, and should ultimately result in genomic selection models with higher predictive abilities.

In general, lower prediction accuracies were obtained for the biomass quality traits relative to the morphology traits. We suspect that this result could have arisen from two different sources. In contrast to the morphological traits, the process of obtaining the quality traits was a lengthy procedure that was conducted in the laboratory. As such, it is possible that a greater amount of variability was introduced into the quality traits, which ultimately resulted in lower prediction accuracies. Factors such as spatial variability in the field, diurnal variation in biomass quality traits manifested by variation in sampling time, variation in grinding time and blade sharpness, and moisture content of the samples may all introduce variability to the measurement of biomass quality traits.

Our study suggests that the implementation of genomic selection approaches to switchgrass breeding programs will be highly beneficial. We believe that such an approach will revolutionize switchgrass breeding programs just as it has in at least four dairy cattle breeding programs [17]. Indeed, the large body of theoretical and empirical studies conducted in plant and animal species [15], [22], [25], [46], [48], [49] suggests that genomic selection is a cost-effective approach that will substantially speed up breeding cycles, and we expect that these advantages will significantly benefit the development of switchgrass as a biofuel feedstock. As high as the prediction accuracies were in our study, we expect them to increase as more attention is focused on the characterization and exploitation of switchgrass phenotypic and genotypic resources. Specifically, we believe that increased prediction accuracies will arise from improvements to the switchgrass reference genome, improvements in phenotyping techniques, and the development of markers with higher levels of genomic coverage and density.

## Supporting Information

Table S1 **Correlation matrix for untransformed BLUPs of the seven morphological traits.** Pearson correlation coefficients are presented in the upper triangle, and the P-values for the significance of associations are in the lower triangle.
(XLS)

Table S2 **Correlation matrix for untransformed BLUPs of the 13 quality traits.** Pearson correlation coefficients are presented in the upper triangle, and the P-values for the significance of associations are in the lower triangle.
(XLS)

File S1 **Data files used to conduct analysis.** All files used to conduct the genomic selection analysis are included in this file.
(ZIP)
